# Size‐Controlled Boron‐Based Bifunctional Photocathodes for High‐Efficiency Photo‐Assisted Li–O_2_ Batteries

**DOI:** 10.1002/advs.202301682

**Published:** 2023-05-17

**Authors:** Ling Li, Fuquan Ma, Congying Jia, Qi Li, Xuexia He, Jie Sun, Ruibin Jiang, Zhibin Lei, Zong‐Huai Liu

**Affiliations:** ^1^ Key Laboratory of Applied Surface and Colloid Chemistry (Shaanxi Normal University) Ministry of Education Xi'an 710062 P. R. China; ^2^ Shaanxi Key Laboratory for Advanced Energy Devices Shaanxi Normal University Xi'an 710119 P. R. China; ^3^ School of Materials Science and Engineering Shaanxi Normal University Xi'an 710119 P. R. China

**Keywords:** bifunctional photocathodes, boron, extra‐long durability, Li–O_2_ batteries, round‐trip efficiencies

## Abstract

Photo‐assisted Li–O_2_ batteries are introduced as a promising strategy for reducing severe overpotential by directly employing photocathodes. Herein, a series of size‐controlled single‐element boron photocatalysts are prepared by the meticulous liquid phase thinning methods by combining probe and water bath sonication, and their bifunctional photocathodes in the photo‐assisted Li–O_2_ batteries are systematically investigated. The boron‐based Li–O_2_ batteries have shown incremental round‐trip efficiencies as the sized reduction of boron under illumination. It is noteworthy that the completely amorphous boron nanosheets (B_4_) photocathode not only delivers an optimizing round‐trip efficiency of 190% on the basis of the ultra‐high discharge voltage (3.55 V) and ultra‐low charge voltage (1.87 V) but also gives a high rate performance and ultralong durability with a round‐trip efficiency of 133% after 100 cycles (200 h) compared with the other‐sized boron photocathodes. This remarkable photoelectric performance of the B_4_ sample can be attracted to the synergistic effect on the suitable semiconductor property, high conductivity, and strengthened catalytic ability of boron nanosheets coated with ultrathin amorphous boron‐oxides overlayer. This research can open a new avenue to facilitate the rapid development of high‐efficiency photo‐assisted Li–O_2_ batteries.

## Graphical abstract

Boron‐based photo‐assisted Li–O_2_ batteries are systematically investigated for the first time, and experiments show excellent round‐trip efficiency and ultralong durability. This study demonstrates that single‐element boron‐based materials can be used as a bifunctional photocatalyst in photo‐assisted Li–O_2_ batteries, and such a system with single‐element photoelectrode may open a promising pathway for high‐efficiency photoelectric conversion and storage in Li–O_2_ batteries.

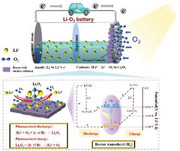

## 1 Introduction

Driven by the increasing requirements of energy storage/conversion systems, the rechargeable lithium–oxygen (Li–O_2_) batteries have been considered as one of the most promising candidates due to their ultra‐high theoretical energy storage density.^[^ ^1^ ^]^ The typical Li–O_2_ batteries belong to the metal–air batteries with lithium metal anode and oxygen active cathode via the formation and decomposition of solid Li_2_O_2_ corresponding to the oxygen reduction reaction (ORR) and oxygen evolution reaction (OER) in the cathode (2Li^+^ + 2e^−^ + O_2_ ↔ Li_2_O_2_, E*^*θ*^*  = 2.96 V).^[^ ^2^ ^]^ However, the sluggish ORR and OER dynamics, the poor conductivity and insolubility of the discharge product Li_2_O_2_, and the unsatisfactory electrode structure have resulted in severe overpotential, even the low rate and cycle life for Li–O_2_ batteries.^[^ ^3^ ^]^ The photo‐assisted Li–O_2_ batteries have been proven to be an effective strategy to reduce the overpotential by directly introducing the photocathode into Li–O_2_ batteries.^[^ ^4^ ^]^ Suitable photocatalysts have emerged as the crucial factor in this photo‐assisted Li–O_2_ battery system, and have been expected to preferably have a wide light‐absorption range, high charge separation efficiency, fast transfer of charge, and abundant catalytic sites. Currently, extensive studies have shown that some inorganic/organic compounds photocatalysts have presented the utilized potentials, such as TiO_2_, C_3_N_4_, WO_3_, ZnS@CNT, Ag/Bi_2_MoO_6_, *α*‐Fe_2_O_3_‐NiOOH, siloxene, Co‐TABQ, and (4,4′‐EDP) Pb_2_Br_6_ photocathodes.^[^ ^4^, ^5^ ^]^ While the single‐element photocathodes have been rarely reported for utilization in this system, much less the storage mechanism and prospect forecast.

As the neighbor of carbon in Group IIIA of the element periodic table, the sole nonmetal element boron (B) has been found to own the electron‐deficient nature due to the (He)2s^2^2p^1^ ground‐state electron structure with valence electron number 3, while atomic orbital number 4.^[^ ^6^ ^]^ The peculiar electronic structure and bonding modes determine the polyhedral feature of boron, which contribute to the formation of complex polyhedral structures obviously distinguishing from the one‐layered structure of graphene.^[^ ^7^ ^]^ At least 17 allotropes of boron have been studied so far, all of them are bounded in a different way by using B_12_ icosahedron as their fundamental structural unit.^[^ ^8^ ^]^ Based on its special electron‐deficient nature and complex structure, boron has shown many special properties including exceptional optical, electronic, chemical, and mechanical properties.^[^ ^9^ ^]^ In especial, the semiconductivity and structural stability of boron could determine the application potential of this photocatalyst in photo‐assisted Li–O_2_ batteries. At present, the semiconductivity of several phases of boron has been investigated experimentally. For instance, Zeng et al.^[^ ^10^ ^]^ have reported that *α*/*α*′‐typed boron sheets are assigned to the small‐gap semiconductors with indirect bandgaps of 1.4/1.10 eV. Cheng et al.^[^ ^11^ ^]^ have indicated that the *β*‐rhombohedral boron with a higher bandgap of 1.5–1.6 eV delivers the photocatalytic activity in generating ⋅OH radicals under irradiation, suggesting that the development of nanometer‐sized boron could lead to high photoactivity in the future. Ye et al.^[^ ^12^ ^]^ have demonstrated that the amorphous boron powder containing traces of crystalline *β*‐rhombohedral boron enables the direct and efficient reduction of CO_2_ into CO and CH_4_ in the presence of water and under light irradiation. These experimental results indicate that some suitable boron phases with different sizes and morphologies maybe are good photocatalysts for photo‐assisted Li–O_2_ batteries.

In this work, the size‐controlled boron‐based materials (recorded from B_1_ to B_5_ along with the progressive decrease in sizes) have been prepared by the meticulous liquid phase thinning methods by combining probe and water bath sonication, and they have been explored as the bifunctional photocathodes in the photo‐assisted Li–O_2_ batteries systematically. Especially, the completely amorphous boron nanosheet (B_4_) has been prepared successfully for the first time, and it exhibits an ultra‐high discharge voltage (3.55 V) and ultra‐low charge voltage (1.87 V) with an optimizing round‐trip efficiency of 190% in the photo‐assisted Li–O_2_ batteries, which outperforms the other‐sized boron samples and most reports with other photocatalysts. Although the quantum size effect of boron photocatalysts has gradually emerged with dimensional attenuation, the boron quantum dots could be more easily oxidized and form the crystalline boron oxide concentrated layer with a low conductivity during the preparation process. Thus, the befitting semiconductor characteristic from the completely amorphous boron nanosheet and the strengthened catalytic capacity of amorphous boron oxides overlayer could produce the synergistic effect to account for the high‐efficiency of B_4_‐based photo‐assisted Li–O_2_ batteries. This research could highlight the preparation significance of various‐sized boron materials and provide a new avenue to facilitate the rapid development of the photo‐assisted Li–O_2_ batteries.

## 2 Results and Discussions

### 2.1 Preparation and Characterization of the Boron Samples

**Figure 1:**
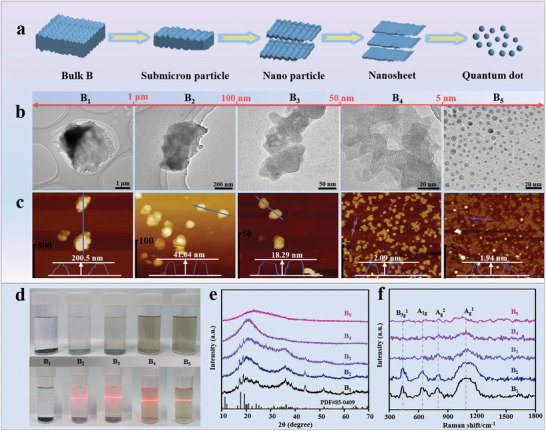
Structure and morphology characterization of size‐controlled boron materials: a) preparation illustration of boron with different sizes, b,c) TEM and AFM images, d) photo‐images of various‐sized boron samples dispersed in acetonitrile solvent, e) XRD patterns, and f) Raman spectra.

Different‐sized boron samples are obtained by a specific stripping of bulk boron in acetonitrile solvent with the tunable probe, water bath ultrasound, and subsequent differential centrifugations, as detailed in **Figure** **1**a and Figure S1 (Supporting Information), respectively. As‐prepared boron samples range from micron to nanometer scales, including bulk boron (B_1_), submicron particle boron (B_2_), nanoparticle boron (B_3_), nanosheet boron (B_4_) and quantum dot boron (B_5_). The scanning electron microscopy (SEM) images give a clear transformation tendency of boron samples from the micron to nanometer scale (Figure S2, Supporting Information). To better confirm the planar size and thickness evolutions of prepared boron samples, the structure morphologies of boron samples are confirmed by transmission electron microscopy (TEM) and atomic force microscopy (AFM). As shown in Figure 1b,c, the size of bulk B_1_ is larger than 1 µm, and its thickness is ≈200 nm. However, the average planar sizes of the boron samples (B_1_‐B_5_) gradually decreased from ≈2.1 µm to ≈0.9 µm, ≈73.3 nm, ≈8.7 nm, and ≈3.6 nm

(Figure S3, Supporting Information) and the average thickness also decreased from ≈201 nm of B_1_ to 41, 18.3, 2.09, and 1.94 nm after the combined action of the probe ultrasound and water bath ultrasound and the centrifugal separation processes. Figure 1d shows the dispersion photographs of the different‐sized boron samples in acetonitrile solvent for 48 h, and the dispersed concentrations of boron samples in acetonitrile solvent gradually increase as the boron size decreased especially for the B_4_ and B_5_ samples, and a Tyndall effect becomes more and more pronounced. In particular, the boron nanosheet (B_4_) and boron quantum dot (B_5_) have still maintained the high‐concentrated dispersion in acetonitrile solvent after 48 h and formed the brown colloidal solution with a more obvious Tyndall‐effect, demonstrating that the high‐quality nanosheets and quantum dots have been prepared successfully. Moreover, with the benefit of obtaining boron samples of significantly different sizes, the effect of size on the sorption properties can be explored. The N_2_ adsorption/desorption isotherms are shown in Figure S4 (Supporting Information). The overall N_2_ uptake is observed to increase with increasing size from ≈2 µm to 2 nm for different‐sized boron samples. This is due to the size effect of boron samples which gradually emerges as the dimensional attenuation, leading to an increase in its specific surface area.^[^ ^13^ ^]^ Then, the crystal structures of the different‐sized boron samples are characterized by X‐ray diffraction (XRD) and Raman spectra. From the XRD patterns (Figure 1e), it can be seen that bulk B_1_ sample could be indexed to the weak crystalline *β*‐rhombohedral boron (PDF#85‐0409), and the crystal diffraction peaks gradually weaken as the sizes of boron samples gradually reduce. Significantly, the crystal diffraction peaks are difficultly observed, and the retained halo peaks indicated that B_4_ and B_5_ samples could mainly display the amorphous states. The corresponding phenomenon is also detected in their Raman spectra (Figure 1f). The peaks of bulk boron located at 435.3, 637.4, 804.1, and 1149.5 cm^−1^ are assigned to the B_3g_^1^, A_1g_, A_g_^2^, and A_g_^1^ vibration modes respectively,^[^ ^14^ ^]^ and their corresponding peak intensity has decreased and widened with the decrease of boron sizes, suggesting the crystal structure of boron samples presents an attenuated size‐effect, and the amorphous phase has been formed as the boron sizes are reduced to the nanometer. Besides, the TEM and corresponding fast fourier transformation images also have confirmed that the structures of boron samples have evolved from the weaken crystalline to amorphous states (Figure S5, Supporting Information). But the remarkable thing is that the clear lattice fringes assigned to B_2_O could be observed for the B_5_ sample, and this result has indicated that the polyactive‐sited surface of the boron quantum dot could be more easily oxidized for forming the crystalline boron‐oxide overlayer, which was difficultly detected by the XRD characterization due to the quantum‐dot size (Figure S6, Supporting Information).

**Figure 2:**
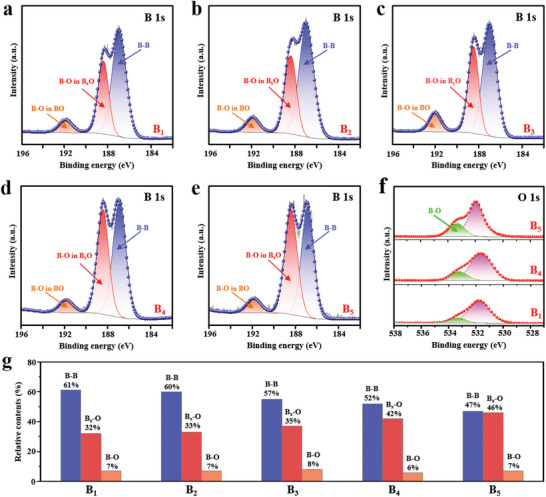
XPS spectra of boron samples with different sizes: a–e) B 1s spectra of B_1_, B_2_, B_3_, B_4_, and B_5_ samples, respectively, f) O 1s spectra of B_1_, B_4_, and B_5_ materials, and g) Relative contents of fitting peak area in corresponding B 1s XPS spectra.

X‐ray photoelectron spectroscopy (XPS) further reveals the influence of liquid thinning on surface compositions and chemical states for the as‐prepared different‐sized boron samples. As can be seen in the high‐resolution B 1s XPS region (**Figure** **2**a–e; Figure S7, Supporting Information), three obvious peaks for all samples (B_1_‐B_5_) are shown. Three peaks resolved at 186.9, 188.4, and 191.8 eV are respectively observed for boron samples, the strong peak at low binding energy is attributed to B—B bonding (186.9 eV), while the higher binding energy components at 188.4 and 191.8 eV are attributed to the oxidation of boron. The component at 188.4 eV is assigned to the B—O bond in a boron‐rich oxide in which an oxygen atom is coordinated with several boron atoms (B_x_O (1 < *x* < 6)), while the highest energy component at 191.8 eV is characteristic of B—O.^[^ ^11^, ^12^, ^15^ ^]^ In contrast to the bulk boron sample (B_1_), the B—O peak relatively increases with decreasing the sizes of the boron grain, revealing the boron oxidation during the preparation of liquid thinning. The O 1s spectrum at 533.2 eV assigned to the B—O bond has shown that the concentrations of the B—O bond have increased with the reduction of the boron samples size, and the B_5_ has the B—O bond has increased as the reduction of boron samples size, and the B_5_ has the highest content ratio of born oxides (Figure 2f). In combination with the fitting peaks of B 1s XPS spectra visually (Figure 2g), it should be noticed that the B_5_ sample could be easily oxidized and form more coating layer of boron oxides due to richer activation sites on the surface of boron quantum dots. The boron oxide coating layer can be explained by the use of an XPS etching treatment, where the boron oxide peaks tend to become weaker with increasing etching time (Figure S8, Supporting Information). When the B_4_ sample is exposed to pure O_2_ atmosphere for 10 h, its XPS spectrum shows that the boron oxide coating layer has reached a saturation concentration in company with preparation in air, and this enriched coating layer can protect the bulk phase boron from the further oxidation (Figure S9, Supporting Information).

### 2.2 Photochemical Performance of Boron‐Based Photo‐Assisted Li–O_2_ Batteries

**Figure 3:**
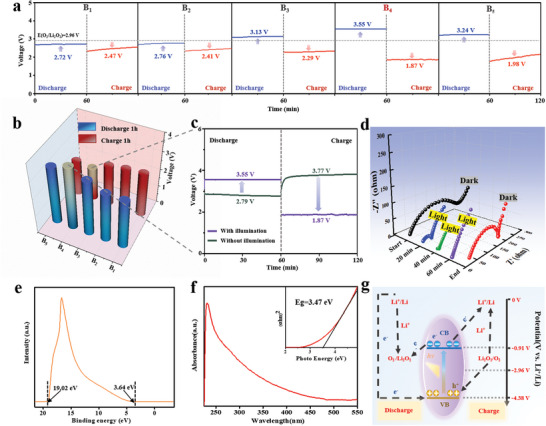
Photoelectric storage performance and working mechanism of photo‐assisted Li–O_2_ batteries employed by the size‐controlled boron photocathodes: a,b) discharge and charge profiles and the corresponding potentials diagram of size‐controlled boron‐based Li–O_2_ batteries at 0.03 mA cm^−2^, c) discharge and charge curves of B_4_‐based Li–O_2_ battery with and without illumination, d) corresponding EIS curves of B_4_‐based Li–O_2_ battery at different times with and without illumination, e,f) UPS and UV spectra of B_4_ sample, and g) the schematic diagram of working potentials for B_4_‐based Li–O_2_ battery.

Photo‐assisted charge/discharge could be one of the most promising strategies for reducing the overpotential and saving the input electric energy. The prepared boron samples with various sizes have been employed as the photocathode indirectly integrated into Li–O_2_ batteries and their photoelectric conversion and storage performance have been systematically evaluated by a series of photochemical measurements with illumination (a 500 W Xe lamp as the simulative light source). The photo‐assisted discharge/charge curves have been achieved for boron‐based Li–O_2_ batteries as shown in **Figure** **3**a. At a current density of 0.03 mA cm^−2^, the all boron‐based Li–O_2_ batteries present a relatively stable potential platform, and the photo‐assisted discharge/charge potentials (V vs Li^+^/Li) of 2.72/2.47, 2.76/2.42, 3.13/2.29, 3.55/1.87, and 3.24/1.98 V have been obtained for corresponding to the B_1_‐B_5_ photocathodes respectively. Among them, the discharge/charge potentials exhibit a progressively increasing/decreasing tendency respectively in company with the size reduction of the used boron photocatalysts, especially an optimal round‐trip efficiency up to 190% is obtained for B_4_ photocathode based on the ratio of discharge and charge potentials under illumination, and it is obviously superior to those of other sized boron photocathodes (Figure 3b). That is related to the fact that smaller particle size leads to a larger specific surface area, which in turn leads to a larger contact area between the material and the electrolyte, thus providing more catalytically active sites to promote the ORR and OER as a Li–O_2_ battery cathode, as well as facilitating the diffusion of oxygen and the electrolyte. However, the B_5_ photocathode delivers a lower round‐trip efficiency of 163% than that of the B_4_ photocathode, it could be caused by the negative effect of more boron oxides coating layer on the surface of boron quantum dots. The corresponding discharge/charge potentials without illumination show a similar pattern the all boron‐based Li–O_2_ batteries (Figure S10, Supporting Information).

For better evaluating the performance of B_4_‐based Li–O_2_ batteries, the discharging and charging performance have been compared with and without illumination (Figure 3c). As a result, the discharge potential has been improved from 2.79 to 3.55 V, and the charge potential has been decreased from 3.77 to 1.87 V due to the optical compensation. The above experiment results reveal that the outstanding performance of B_4_‐based Li–O_2_ batteries could be put down to the synergistic effect of the photo and electrocatalysis from the B_4_ photocathode. The corresponding electrochemical impedance spectroscopy (EIS) curves (Figure 3d)

also indicate that the B_4_ photocathode shows a lower charge transfer resistance and more rapid ion diffusion under light illumination in comparison without illumination in Li–O_2_ batteries. Then, the working mechanism of the B_4_ photocathode is discussed by combining the analysis of semiconductor properties. From the UV–vis absorption spectroscopy spectrum (Figure 3e), the UV photoelectron spectroscopy (UPS) spectrum (Figure 3f) and amperometric I–t curves without a bias voltage (Figure S11, Supporting Information), it can be detected that the B_4_ sample enables to present an extensive absorption in the band from 200 to 550 nm (visible and ultraviolet regions), a band gap (E_g_) of 3.47 eV, the conduction band (CB) and valence band (VB) of 2.37 and 5.48 eV (vs SHE), and a rapid light‐respond and high photocurrent of 2.7 mA cm^−2^ without a bias voltage. During the photo‐assisted discharge, the photoexcited electrons of B_4_ photoelectrode can cause an ORR transition of 2Li^+^ + O_2_ + 2e^−^_(CB)_ → Li_2_O_2_, and the excited holes stayed in VB is combined with the electrons from the external circuit. Moreover, it also can conduct a reversible photo‐assisted charge with an OER process of 2h^+^_(VB)_ + Li_2_O_2_ → 2Li^+^ + O_2_ on the photocathode.

**Figure 4:**
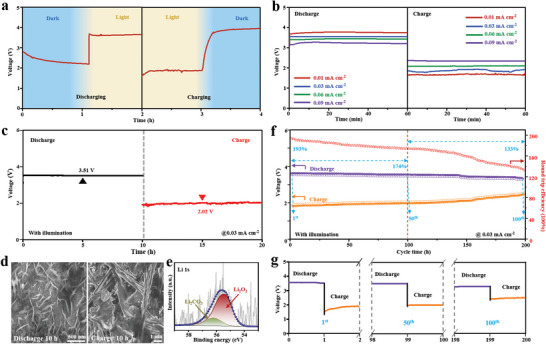
Rate‐performance and durability of photo‐assisted B_4_‐based Li–O_2_ battery: a) light‐responding discharge and charge curves with and without illumination at 0.03 mA cm^−2^, b) discharge and charge curves under illumination at various current densities of 0.01, 0.03, 0.06, and 0.09 mA cm^−2^, c) discharge and charge curves for 10 h with illumination at 0.03 mA cm^−2^, d) SEM images of discharged and charged products after the illumination for 10 h, e) Li 1s XPS spectrum of the discharged product after illumination, f) cycle performance curve after 200 cycles with illumination at 0.03 mA cm^−2^, and g) discharge/charge curves of first, fifth, and 100th cycles.

The stability and durability have been further investigated for B_4_‐based Li–O_2_ batteries under illumination. **Figure** **4**a shows the discharge/charge behaviors of the Li–O_2_ batteries with the B_4_ photocathode responding to alternate light on/off at 0.03 mA cm^−2^. The discharge voltage is rapidly increased from 2.34 V in the dark to 3.61 V under light irradiation, and simultaneously the charge voltage is quickly reduced to 1.89 V under light irradiation from 3.92 V in the dark. The above behaviors have clearly demonstrated that the B_4_ photocathode enables to present the fast light‐respond with the stabilized potential platform under illumination. Besides, the discharge/charge curves have also been obtained at the diversified current densities (Figure 4b). The smooth potential platforms have been recorded as 3.78/1.71, 3.55/1.92, 3.45/2.09, and 3.24/2.34 V at a current density of 0.01, 0.03, 0.06, and 0.09 mA cm^−2^, respectively. The B_4_ photocathode can achieve a high discharging potential passed by 3.2 V and the charging potential still maintains below 2.3 V as the current density increases from 0.01 to 0.09 mA cm^−2^, suggesting the B_4_‐based Li–O_2_ battery can steadily discharge and charge even at high current density and own a good rate capability under illumination. The long‐time discharge/charge curves have been tested for B_4_‐based Li–O_2_ battery. As displayed in Figure 4c, this system can be discharged and charged for 20 h (discharge for 10 h and charge for 10 h), and the discharge/charge potential platforms are able to hold the 3.51/2.02 V, indicating the ultra‐high stable charge‐discharge performance of the assembled boron‐based Li–O_2_ batteries for the long terms. To further observe the morphological changes of substances on the surface of the oxygen cathode during the charge/discharge process, the morphologies of products in different test periods are observed by SEM images (Figure 4d).^[^ ^16^ ^]^ For the pristine B_4_ photocathode, the substance is mainly a boron sample loaded on nickel foam (Figure S12, Supporting Information). The thin leaves‐liked products assigned Li_2_O_2_ are generated on the surface of the discharge photocathode after 10 h, and these products have been confirmed by the Li 1s XPS spectrum (Figure 4e).^[^ ^4^, ^17^ ^]^ While, this product Li_2_O_2_ has been broken down and disappeared for the charged photocathode, reflecting the high reversibility of ORR and OER and the high‐durability of B_4_ photocathode. The cycle performance of the B_4_ photocathode is measured at a small current density of 0.03 mA cm^−2^ (Figure 4f), and the experiment result shows that the round‐trip efficiencies of B_4_‐based Li–O_2_ battery still retain the 174% after 50 cycles (0.38% attenuation rate for 100 h) and 133% after 100 cycles of 200 h (only 0.82% loss for 200 h). From the enlarged charge‐discharge curves for the first, 50th, and 100th cycles, it can be confirmed further that the B_4_‐based Li–O_2_ battery not only possesses the smooth and stabilized potential platforms for the long‐cycling tests but also maintains an ultra‐long cycle life (Figure 4g).

**Figure 5:**
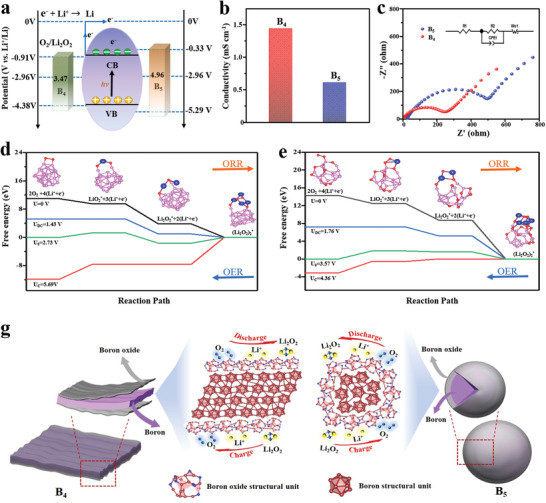
Photoelectric conversion and storage mechanism of B_4_ and B_5_ photocathodes: a–c) working potential diagrams, conductivities, and EIS curves for B_4_ and B_5_ samples respectively, d,e) calculated free‐energy diagrams for the discharge‐charge reactions in the B_28_ and B_20_O_8_‐based Li–O_2_ batteries (purple, red, and blue ball atoms represented B, O, and Li, respectively), and f) structure diagram of B_4_ and B_5_ photocathodes and the corresponding charge‐discharge working mechanism.

In order to elucidate the photoelectric storage regularity of the sized‐controlled boron photocathodes in depth, the working mechanism of B_4_ and B_5_ photocathodes has been compared and analyzed. Through the UPS and UV characterizations (Figure 3e,f; Figures S13,S14, Supporting Information), the theoretical discharge/charge potentials have been estimated as 4.38/0.91 V and 5.29/0.33 V (vs Li^+^/Li) for B_4_ and B_5_ photocatalysts respectively (**Figure** **5**a). B_5_ photocatalyst could predictably possesses higher round‐trip efficiency, nevertheless, the B_4_ photocatalyst exhibits the optimizing ratio of output and input potential platforms practically. To analyze the above reasons, the conductivities and EIS curves of the two photocathodes have been explored (Figure 5b,c). According to the tested results, the conductivity of the B_5_ sample (0.61 mS cm^−1^) is lower than that (1.44 mS cm^−1^) of the B_4_ sample because of more low‐conductive crystalline boron oxide overlayer and higher bandgap for B_5_ sample. Moreover, the B_4_‐based Li–O_2_ battery displays smaller internal charge resistance and faster ion diffusion of 2.3 × 10^−16^ cm^2^ s^−1^, (6.4 × 10^−17^ cm^2^ s^−1^ for B_5_‐based Li–O_2_ battery) (Figures S15,S16, Supporting Information).^[^ ^18^ ^]^ In order to further understand the mechanism of boron and boron oxide in Li–O_2_ batteries, the density functional theory (DFT) calculation of ORR and OER has been performed on the optimized models of B_28_ and B_20_O_8_. The nucleation/decomposition pathway of a Li_4_O_4_ cluster on the catalyst surface is investigated as shown in Figure 5d,e. The discharge plateau of the B_28_ catalyst is estimated as 1.43 V, it was lower than that of 1.76 V for the B_20_O_8_ catalyst, while the charge plateau of B_28_ (5.69 V) is higher than that of B_20_O_8_ (4.36 V) based on the DFT calculation. This result demonstrates that the B_20_O_8_ could facilitates carrier separation, which would make the formation and decomposition of Li_2_O_2_ easy.^[^ ^4^, ^19^ ^]^ Furthermore, the adsorption energy and adsorption structure of the reactant, intermediate, and product on the surface of B_28_ and B_20_O_8_ are also calculated and analyzed (Figure S17, Supporting Information). In comparison with the adsorption energy of oxygen on B_20_O_8_ (E_ads_ = −1.66 eV), the more powerful adsorption for B_28_ surface (E_ads_ = −4.86 eV) has signified that the abundant O_2_. more tends to adhere on the surface of boron, and the boron is more susceptible to oxidation. Figure 5g shows the schematic diagram of both B_4_ and B_5_ photocathodes during the charge‐discharge reaction in Li–O_2_ batteries. Given that the surface of the boron sample and highly active quantum dots are easily oxidized, the B_5_ sample could diver more coating layer of crystalline boron oxides than the B_4_ sample. Thus, the amorphous boron oxides overlayer could play the multifunctional roles that it enables to protect the born bulk from further oxidation, and accelerate the catalytic reaction of OER and ORR of O_2_/Li_2_O_2_, meanwhile, its high conductivity is beneficial to rapid electron transport. Therefore, the suitable semiconductor properties and a thin amorphous coating layer of amorphous boron oxides could all contribute to the excellent performance of B_4_ photocathode in the photoassisted Li–O_2_ batteries.

## 3 Conclusion

In summary, a series of sized‐controlled single‐element boron photocatalysts have been prepared controllably and employed as photocathodes in Li–O_2_ batteries. The boron‐based Li–O_2_ batteries deliver an incremental round‐trip efficiency as the sized reduction of boron under illumination. The B_4_‐based Li–O_2_ battery can display the highest discharge/lowest charge potential of 3.55/1.87 V at 0.03 mA cm^−2^, an ultrahigh round‐trip efficiency of 190% compared with the other size boron samples and most reports with other photocatalysts (Table S1, Supporting Information). Moreover, it shows excellent durability (a round‐trip efficiency of 173% for 20 h continuously) and long‐cycling life (a round‐trip efficiency of 133% after 100 cycles with 200 h). This outstanding photoelectric performance of B_4_‐based Li–O_2_ battery could be put down to the suitable semiconductor property, the electro‐catalytic property, and the ultrathin amorphous boron oxides layer of B_4_ photocatalysts. This amorphous boron oxide overlayer could not only enable to protect the born bulk for further oxidation but also accelerate the catalytic reaction of OER and ORR of O_2_/Li_2_O_2_ and provide high conductivity. These experimental results obviously indicate that element boron‐based materials can be used as a bifunctional photocatalyst in photo‐assisted Li–O_2_ batteries and this designed system with single‐element photoelectrode opens a promising pathway for the high‐efficiency and low‐cost photoelectric conversion and storage in Li–O_2_ batteries.

## Conflict of Interest

The authors declare no conflict of interest.

## Supporting information

Supporting Information

## Data Availability

The data that support the findings of this study are available from the corresponding author upon reasonable request.
