# Supplementary material for: Size‐Controlled Boron‐Based Bifunctional Photocathodes for High‐Efficiency Photo‐Assisted Li–O2 Batteries
Source: Adv Sci (Weinh). 2023 May 17;10(22):2301682. doi: 10.1002/advs.202301682 (PMC10401084; doi:10.1002/advs.202301682)
Supplement: Supplementary file 1 — Supporting Information [file ADVS-10-2301682-s001.pdf]

ADVANCED SCIENCE
Open Access

# Supporting Information

for *Adv. Sci.*, DOI 10.1002/advs.202301682

Size-Controlled Boron-Based Bifunctional Photocathodes for High-Efficiency Photo-Assisted Li–$O_2$ Batteries

*Ling Li, Fuquan Ma, Congying Jia, Qi Li*, Xuexia He, Jie Sun, Ruibin Jiang, Zhibin Lei and Zong-Huai Liu**

**Supporting Information**


**Experimental** ........................................................................................... 4

**Figure S1.** The preparation process of boron samples at different sizes ..................... 5

**Figure S2.** SEM images of boron samples at different sizes ...................................... 6

**Figure S3.** SEM, TEM images and size distributions of boron samples at different sizes. ....................................................................................................... 7

**Figure S4.** $N_2$ adsorption/desorption isotherms of boron samples at different sizes.... 8

**Figure S5.** TEM images (main pictures) and the corresponding FFT images (insets) of boron samples at different sizes ............................................................... 9

**Figure S6.** TEM and HRTEM images of $B_5$ sample ............................................... 10

**Figure S7.** XPS spectra of boron samples at different sizes ..................................... 11

**Figure S8.** B 1s XPS spectra of $B_4$ sample differential etching times ...................... 12

**Figure S9.** B 1s XPS spectra of the $B_4$ sample after 10 h exposure to a pure $O_2$ atmosphere ....................................................................................... 13

**Figure S10.** Discharge and charge profiles and the corresponding potentials diagram of size-controlled boron-based Li-$O_2$ batteries at 0.03 mA $cm^{-2}$ withoutillumination 14

**Figure S11.** Amperometric I-t curves of $B_4$ sample at an applied without a bias voltage under illumination of simulate natural light with 100 s light on/off cycles .. 15

**Figure S12.** SEM image of original $B_4$ photocathode ............................................. 16

**Figure S13.** UPS spectrum of $B_5$ sample ............................................................ 17

**Figure S14.** UV spectrum of $B_5$ sample ............................................................. 18

**Figure S15.** Corresponding faradaic impedance spectra of $B_4$ and $B_5$ samples ............................................................................................... 19

**Figure S16.** Diffusion coefficients derived from the EIS result ................................ 20

**Figure S17.** a) Comparison of the charge density distribution on $B_{28}$ surface. b) Comparison of the charge density distribution on $B_{20}O_8$ surface (the purple, red and bule balls represented B, O, Li, respectively). ............................................... 21

**Table S1.** Comparison of photo-assisted Li-$O_2$ batteries performance with different photocatalysts ................................................................................ 22

**Supplementary References** ........................................................................ 23

## Experimental section

### Chemicals and Materials

Boron powder ( ≥94%) was purchased from Aladdin Reagent. Acetonitrile ($CH_3CN$, AR) and N,N-dimethylformamide (DMF and $C_3H_7NO$, AR) were purchased from Sinopharm Chemical Reagent Co., Ltd. Lithium bis (trifluoromethane sulfonimide) (1.0 M LiTFSI in TETRAGLYME, 100 Vol%) was purchased from DodoChem. All chemicals were of analytical grade and used without further purification.

### Preparation of various-sized boron samples

The liquid phase stripping method has been improved by combining the probe and water bath sonication with a differential centralization technique to prepare boron samples with different sizes from the micron to nanometer level. Detailed operation as follows. Firstly, 120 mg bulk boron powder was grind for 30 min ($B_1$), and then directly added into 60 mL acetonitrile solvent to form the suspension with an initial concentration of 2 mg/mL. Next, the suspension was treated by the probe sonication at a power of 400 W for 6 h with the sonication of 2 s and pause of 3 s. To obtain different-sized boron samples, the as prepared B/acetonitrile solution was carried out by different centrifugation rates and water bath sonication processes. Specifically, the B/acetonitrile suspension was firstly centrifuged at a low speed of 1500 rpm for 10 min to obtain the sediment ($B_2$) and supernatant, and subsequently the supernatant was centrifuged at a high speed of 6000 rpm for 20 min to obtain another sediment ($B_3$) and supernatant ($B_4$). It is worth noting that the preparation of boron quantum dots is slightly different from the above methods. First, the boron powder wasn't required for grinding treatment, the suspension of B/acetonitrile after probing ultrasound for 6 h was centrifuged at 6000 rpm for 20 min and followed by treating by bath sonication for 1 h, boron quantum dots ($B_5$) was finally prepared. Noted that both the probe and water bath sonication processes were conducted under a constant temperature of 0~5 ℃. All kind of products were followed by drying in vacuum at 50 ℃ for overnight to obtain the final sample.

### Materials characterization

The crystallographic structures were characterized by using powder X-ray diffractometer (XRD, Rigaku D/Max-3c, Cu Kα radiation with $\lambda = 1.5406$ Å) with an operating voltage and current of 40 kV and 15 mA. The Raman spectra were measured by using a Renishaw at 532 nm excitation wavelength. The morphologies and nanostructures of boron samples were characterized via a field-emission scanning electron microscopy (FESEM, SU8020), and a transmission electron microscopy (TEM, Tecnai G2 F20) at an acceleration voltage of 200 kV. The thickness and size of the as-prepared boron samples were measured with an atomic force microscope (AFM, Dimension FastScan) in tapping mode under atmospheric conditions. The composition and chemical state were determined by XPS (PHI Quantera II) instrument, and the binding energy calibration was referenced to C 1s at 284.8 eV. An ultraviolet-visible spectrophotometer (UV-vis, UV-3600) was applied for characterizing the absorption spectra of the samples at room temperature.

**Assembling and testing of the Li-$O_2$ batteries**

As-prepared boron products, Super-P, and polyvinylidene fluoride (PVDF) were mixed together at 8:1:1 weight ratio, grinded for 30 min to blend uniformly, and then coated on a clean nickel foams with a diameter of 13 mm, dried in a vacuum oven at 80 ℃ for overnight. The mass loading of active materials per electrode was 10 $mg/cm^{-2}$. CR2032 coin cells with holes on the cathode side were used for testing Li-$O_2$ batteries. The batteries were assembled in a glove box and filled at Ar atmosphere with the moisture and oxygen content of below 0.1 ppm. Li foils were used as the counter, boron cathode was as an oxygen electrode and photoelectrode, and glass fiber (Waterman, GF/A) was applied as the separators. 1.0 M LiTFSI in tetraethylene glycol dimethyl ether (Tetraglyme) was employed as the electrolyte. The assembled cells were stored in a volume capacity of 200 mL sealed glass test device, which was filled with oxygen. Significantly, the oxygen filled glass test devices were purged with oxygen for a few minutes to remove residual gases before being used. Finally, all of the cells were tested on a CHI 760E electrochemical workstation (Shanghai, China). The GEL S500/350 Xe-

lamp (CEAULIGHT, BEIJING) was utilized as the solar source for illumination, and the power was fixed at 500 W.

**Theoretical calculation methods**

The Vienna Ab Initio Package (VASP) had been employed to perform all spin-polarized density functional theory (DFT) calculations within the generalized gradient approximation (GGA) using the Perdew-Burke-Ernzerhof (PBE) formulation. The projected augmented wave (PAW) potentials had been chosen to describe the ionic cores and take valence electrons into account using a plane wave basis set with a kinetic energy cutoff of 500 eV. Partial occupancies of the Kohn-Sham orbitals were allowed to use the Gaussian smearing method and a width of 0.2 eV. The electronic energy was considered self-consistent when the energy change was smaller than $10^{-5}$ eV. The Brillouin zone was integrated with a Monkhorst-Pack 3×3×1 k-point grid for B slab. And, only the Γ point was used to sample the first Brillouin zone for $B_{28}$ clusters and $B_{20}O_8$ clusters. A geometry optimization was considered convergent when the energy change was smaller than 0.02 eV $Å^{-1}$. The weak interaction was described by DFT+D3 method using empirical correction in Grimme's scheme. The Gibbs free energy for each elementary step was calculated as:

$$G = E_{elec} + E_{ZPE} - TS$$

in which $E_{elec}$ was the electronic energy at 0 K calculated by DFT, $E_{ZPE}$ was the zero-point energy term, T was the absolute temperature (here 298.15 K), and S was the entropy.

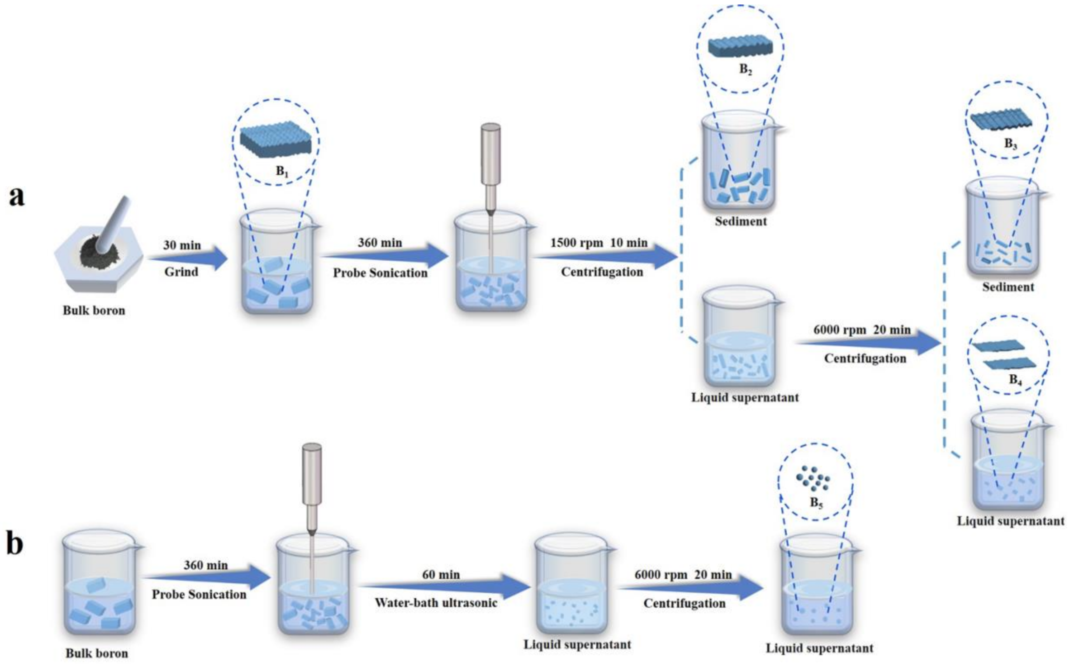


**Figure S1.** The preparation process of boron samples at different sizes.

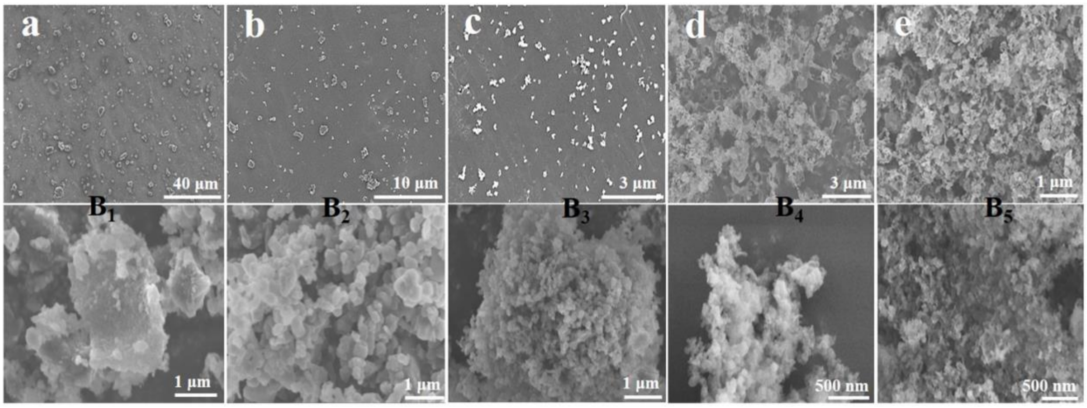


**Figure S2**. SEM images of boron samples at different sizes.

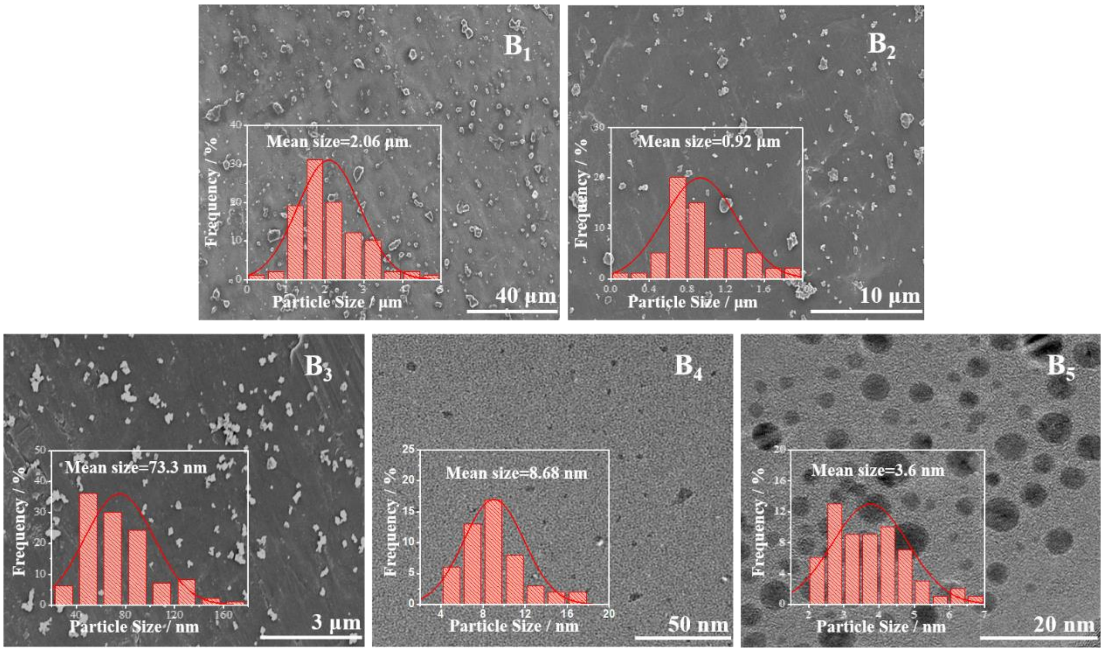


**Figure S3**. SEM, TEM images and size distributions of boron samples at different sizes.

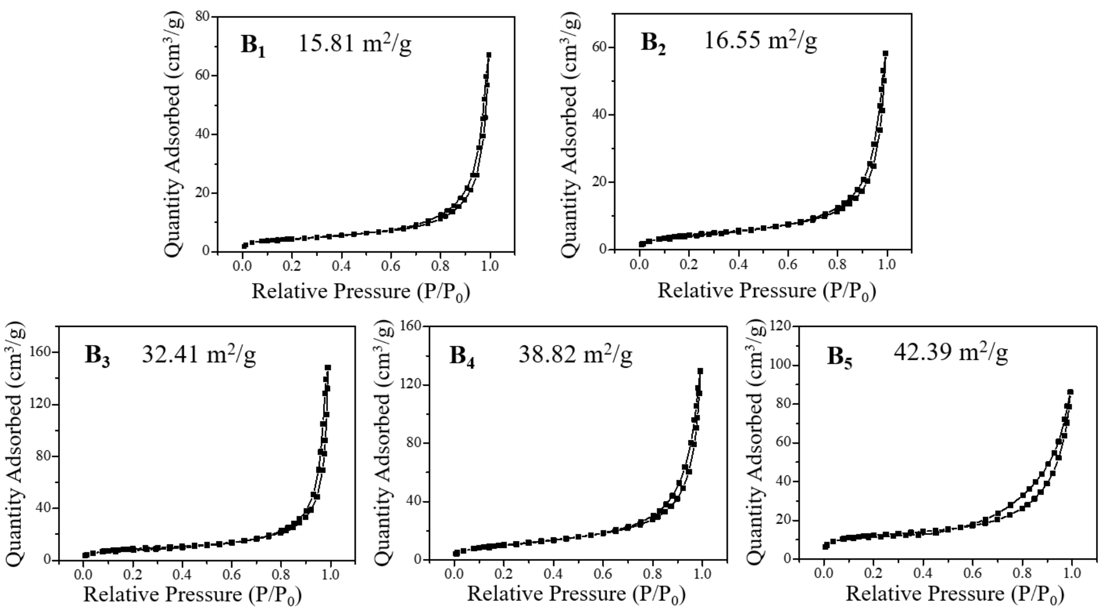


**Figure S4**. $N_2$ adsorption/desorption isotherms of boron samples at different sizes.

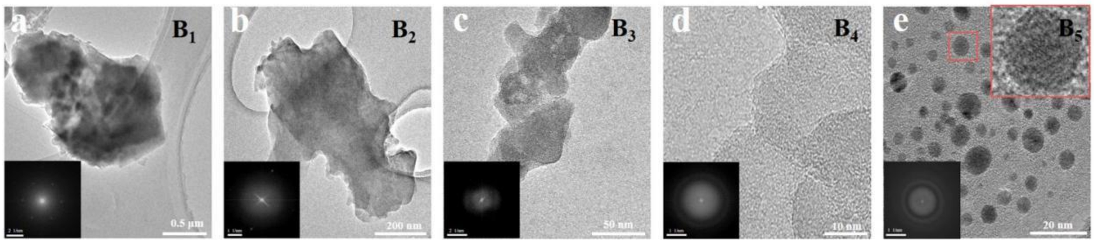


**Figure S5**. TEM images (main pictures) and the corresponding FFT images (insets) of boron samples at different sizes.

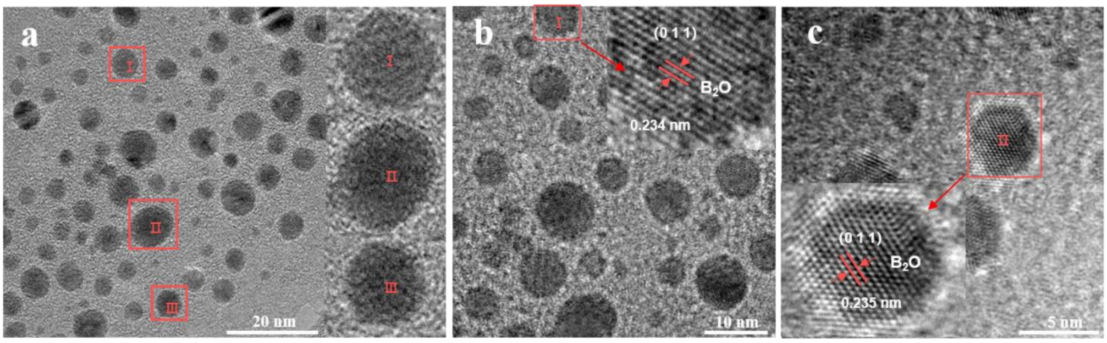


**Figure S6**. TEM and HRTEM images of $B_5$ sample.

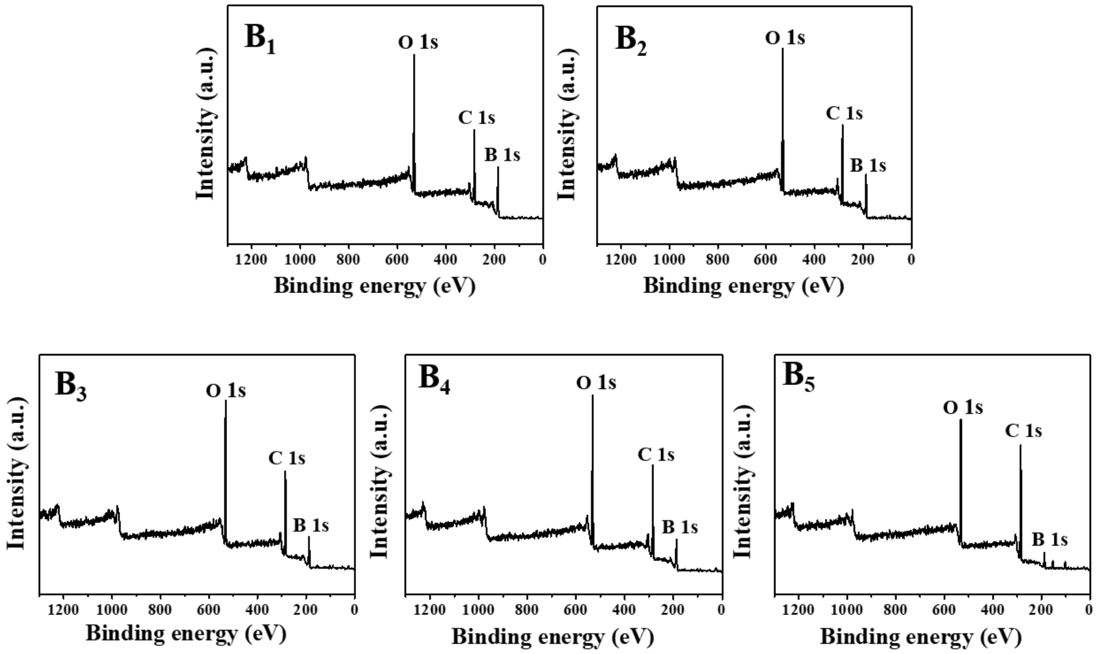


**Figure S7**. XPS spectra of boron samples at different sizes.

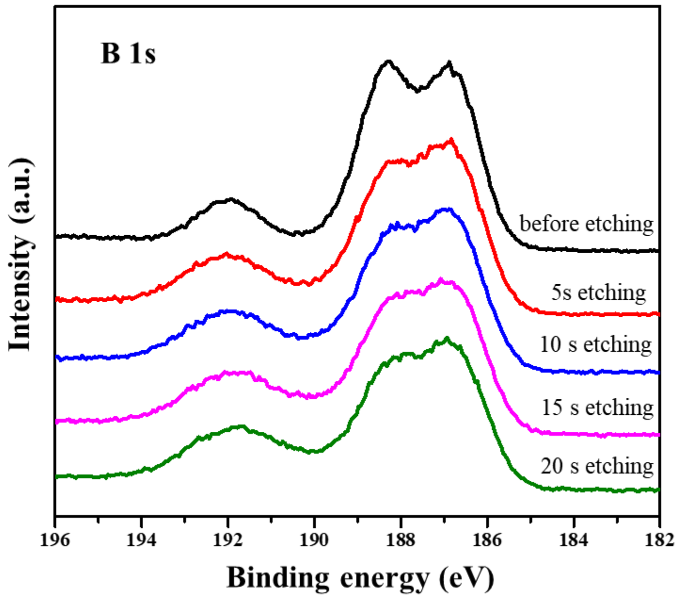


**Figure S8**. B 1s XPS spectra of $B_4$ sample with differential etching times.

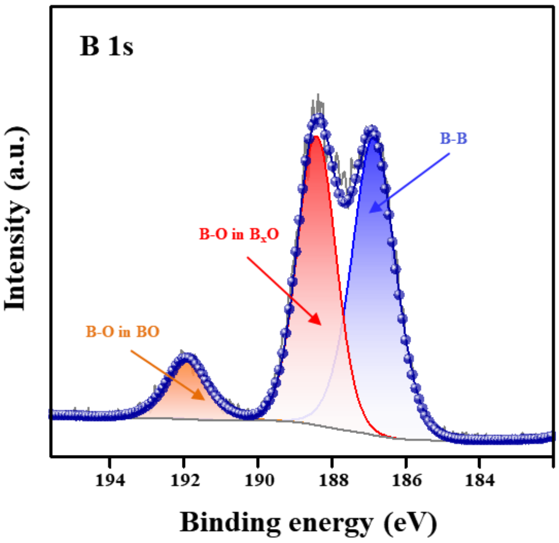


**Figure S9**. B 1s XPS spectrum of the $B_4$ sample after 10 h exposure to a pure $O_2$ atmosphere.

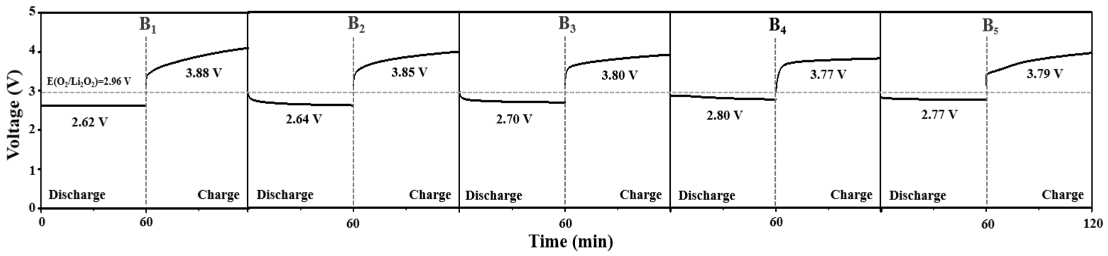


**Figure S10.** Discharge and charge profiles and the corresponding potentials diagram of size-controlled boron-based Li-$O_2$ batteries at 0.03 mA $cm^{-2}$ without illumination.

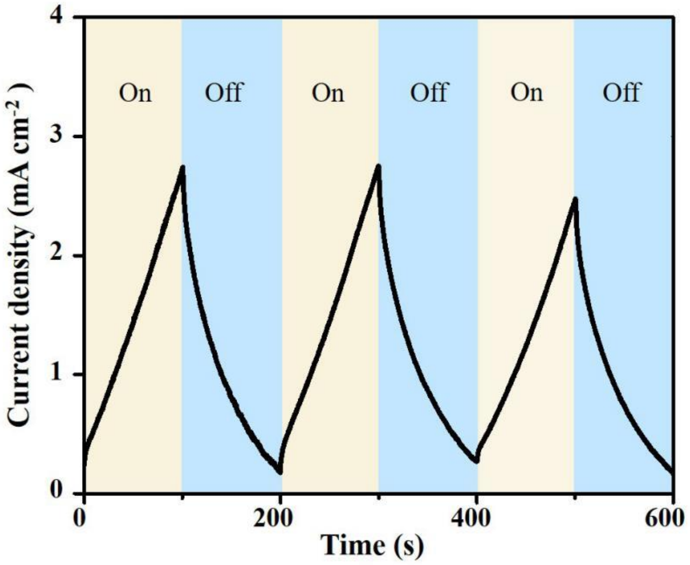


**Figure S11.** Amperometric I-t curves of $B_4$ sample at an applied without a bias voltage under illumination of simulate natural light with 100 s light on/off cycles.

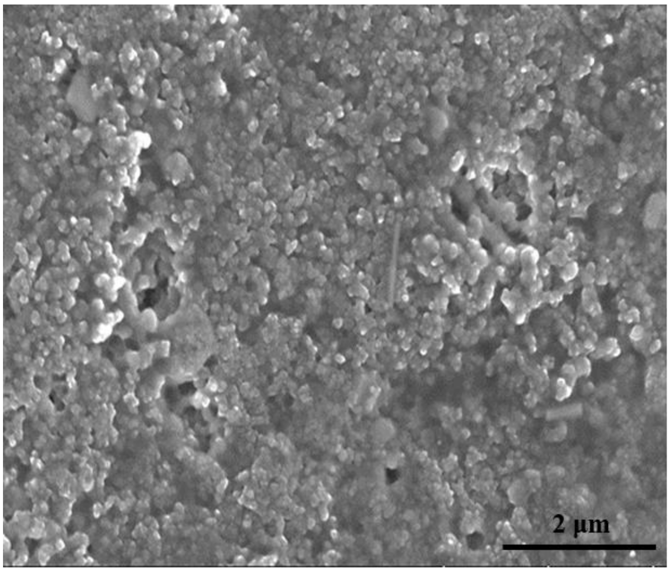


**Figure S12.** SEM image of original $B_4$ photocathode.

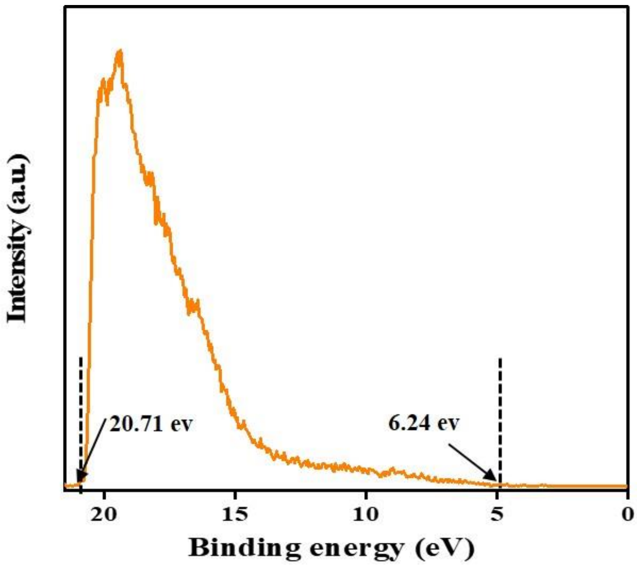


**Figure S13.** UPS spectrum of $B_5$ sample.

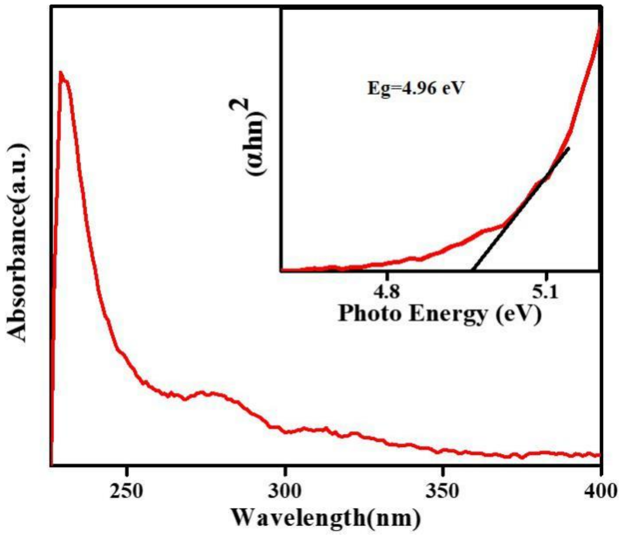


**Figure S14.** UV spectrum of $B_5$ sample.

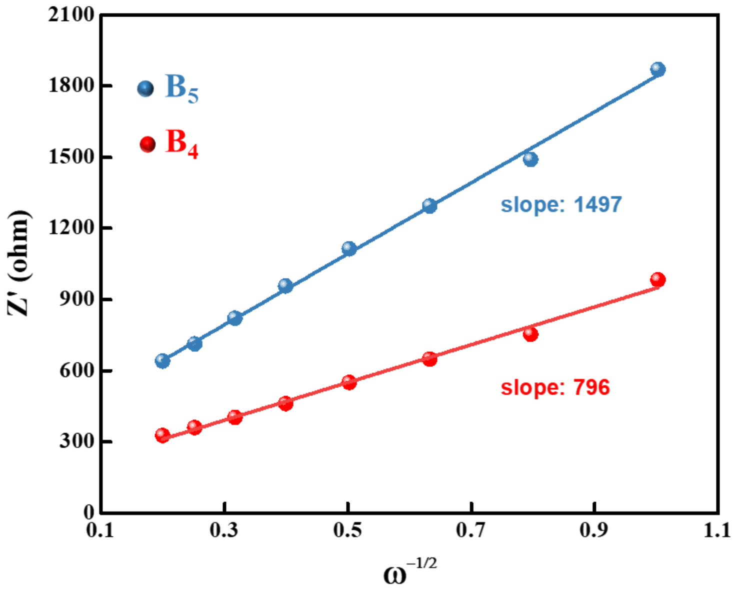


**Figure S15.** Corresponding faradaic impedance spectra of $B_4$ and $B_5$ samples.

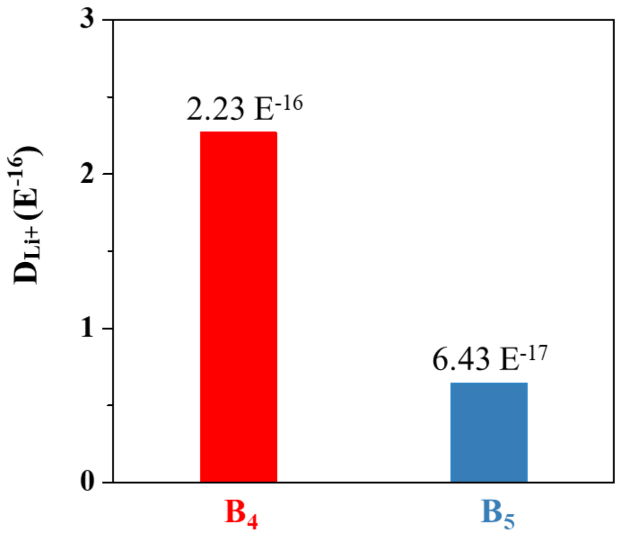


**Figure S16.** Diffusion coefficients derived from the EIS result.

EIS was performed to investigate the kinetics of $Li^+$ diffusion process in the electrode. The $Li^+$ diffusion coefficients were obtained based on the Nyquist plots. The $Li^+$ diffusion coefficient can be calculated using the following equation:

$$D = \frac{R^2T^2}{2A^2n^4F^4C^2\sigma^2} \tag{1}$$

where R is the gas constant with value 8.314 J $K^{-1}$ $mol^{-1}$, T is the room Kelvin temperature of 298 K, A is the electrode area, which is 0.149 $cm^2$, n is the number of the electrons per 2 molecule attending the charge and discharge process, F is the faraday constant (96500 C $mol^{-1}$), C is the concentration of lithium ion of $Li_2O_2$ in boron electrode, which is 0.0263 mol $cm^{-3}$, σ is the slope of the line Z'-$\omega^{-1/2}$, $B_4$ and $B_5$ are 796 and 1497, respectively (Figure S15).

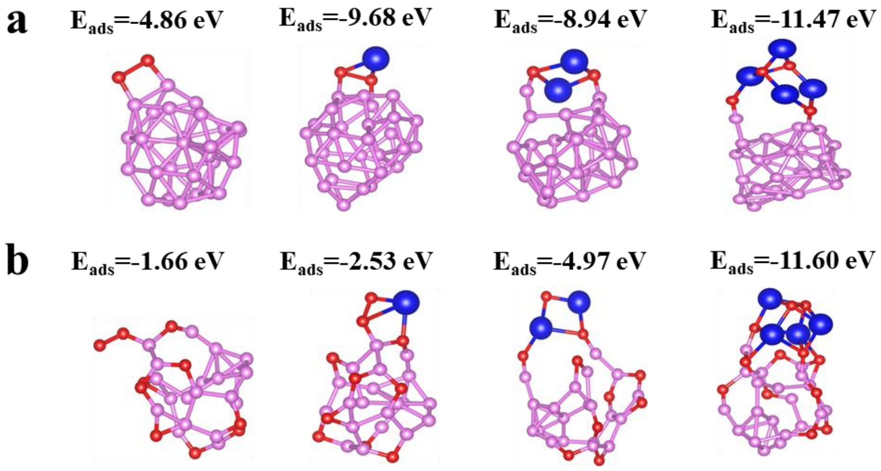


**Figure S17.** a) Comparison of the charge density distribution on $B_{28}$ surface, and b) comparison of the charge density distribution on $B_{20}O_8$ surface (the purple, red and blue balls represented B, O, Li, respectively).

**Table S1.** Comparison of photo-assisted Li-$O_2$ batteries performance with different photocatalysts

| Photocathode catalyst | Current density (mA cm$^{-2}$) | Discharge voltage (V) (vs. Li/Li$^+$ ) | Charge voltage (V) (vs. Li/Li$^+$ ) | Round-trip efficiency (%) | Cycling time (h) | References |
|---|---|---|---|---|---|---|
| Boron ($B_4$) | 0.03 | 3.55 | 1.87 | 190% | 200 | This study |
| Siloxene NSs | 0.075 | 3.51 | 1.9 | 185% | 200 | [1] |
| g-$C_3N_4$/CP+$I^-$/$I_3^-$ | 0.01 | 2.7 | 1.9 | 142% | 100 | [2] |
| pTTh+$I^-$/$I_3^-$ | 0.02 | 3.1 | 3.19 | 97% | 300 | [3] |
| $WO_3$@g-$C_3N_4$ NWA | 0.1 | 2.76 | 3.69 | 75% | 200 | [4] |
| dye-sensitized $TiO_2$ | 0.016 | ~2.8 | 2.76 | ~103% | 50 | [5] |
| TNAs-AuNPs | 0.025 | 2.67 | 2.67 | 100% | 400 | [6] |
| α-$Fe_2O_3$-NiOOH | 0.12 | 2.66 | 3.03 | 88% | 350 | [7] |
| $TiO_2$-$Fe_2O_3$ | 0.01 | 3.01 | 3.2 | 94% | 200 | [8] |
| Co-TABQ | 0.1 | 3.32 | 3.12 | 94% | 100 | [9] |
| $CeVO_4$/CNT | 0.1 | 2.96 | 3.48 | 85% | 100 | [10] |
| ZnS@CNT | 0.026 | 2.35 | 2.08 | 113% | 100 | [11] |
| $TiO_2$/CT | 0.02 | 2.65 | 2.86 | 93% | 60 | [12] |
| Ag-NPs | 0.1 | 3.22 | 3.25 | 99% | 400 | [13] |
| Au/Nv-$C_3N_4$ | 0.05 | 3.16 | 3.26 | 97% | 100 | [14] |
| $C_3N_4$ | 0.04 | 3.22 | 3.38 | 95% | 20 | [15] |
| $LaFeO_3$ | 0.02 | 3.24 | 3.48 | 93% | 400 | [16] |
| $CsPbBr_3$@PCN -333(Fe) | 0.01 | 3.44 | 3.19 | 93% | 200 | [17] |
| (4,4'-EDP) $Pb_2Br_6$ | 0.01 | 2.91 | 3.42 | 85% | 170 | [18] |
| Ag/$Bi_2MoO_6$ | 0.05 A g$^{-1}$ | 3.05 | 3.25 | 94% | 520 | [19] |
| NiO/FNi | 0.01 | 2.63 | 2.92 | 90% | 120 | [20] |

## Supplementary References


[1] C. Y. Jia, F. Zhang, L. N. She, Q. Li, X. X. He, J. Sun, Z. B. Lei, Z. H. Liu, *Angew. Chem. Int. Ed.* **2021**, *133*, 11357.

[2] Y. Liu, N. Li, S. C. Wu, K. M Liao, K. Zhu, J. Yia, H. S. Zhou, *Energy Environ. Sci.* **2015**, *8*, 2664.

[3] W.W. Liu, Y. T. Yang, X. Hu, Q. M. Zhang, C. Y. Wang, J. P. Wei, Z. J. Xie, Z. Zhou, *Small* **2022**, *18*, 2200334.

[4] H. R. Xue, T. Wang, Y. Y. Feng, H. Gong, X. L. Fan, B. Gao, Y. L. Kong, C. Jiang, S. T. Zhang, X. L. Huang Ji. P. He, *Nanoscale* **2020**, *12*, 18742.

[5] M. Z. Yu, X. D. Ren, L. Ma, Y. Y. Wu, *Nat. Commun.* **2014**, *5*, 1.

[6] S. F. Tong, C. P. Luo, J. D. Li, Z. W. Mei, M. M. Wu, A. P. O'Mullane, H. Y. Zhu, *Angew. Chem. Int. Ed.* **2020**, *59*, 20909.

[7] H. Gong, H. R. Xue, B. Gao, Y. Li, X. Y. Yu, X. L. Fan, S. T. Zhang, T. Wang, J. P. He, *ChemComm.* **2020**, *56*, 13642.

[8] M. L. Li, X. X. Wang, F. Li , L. Y. Zheng, J. J. Xu, J. H Yu, *Adv. Mater.* **2020**, *32*, 1907098.

[9] Q. L. Lv, Z. Zhu, S. Zhao, L. B. Wang, Q. Zhao, F. J. Li, L. A. Archer, J. Chen, *J. Am. Chem. Soc.* **2021**, *143*, 1941.

[10] D. W. Li, X. Y. Lang, Y. Guo, Y. Q. Wang, Y. Y. Wang, H. C. Shi, S. C.Wu, W. C. Wang, Q. H. Yang, *Nano Energy* **2021**, *85*, 105966.

[11] Y. Liu, J. Yi, Y. Qiao, D. Wang, P. He, Q. Li, S. C. Wu, H. S. Zhou, *Energy Stor. Mater.* **2018**, *11*, 170.

[12] H. Gong, T. Wang, H. R. Xue, X. L Fan, B. Gao, H. B. Zhang, Li Shi, J. P. He, J. H. Ye, *Energy Stor. Mater.* **2018**, *13*, 49.

[13] L. J. Zheng, F. Li, L. N. Song, M. L. Li, X. X. Wang, J. J. Xu, *Energy Stor. Mater.* **2021**, *42*, 618.

[14] Z. Zhu, Y. X. Ni, Q. L. Lv, J. R. Geng, W. Xie, F. J. Li, J. Chen, *Proc Natl Acad Sci.* **2021**, *118*, e2024619118.

[15] Z. Zhu, X. M. Shi, G. L. Fan, F. J. Li, J. Chen, *Angew. Chem. Int. Ed.* **2019**, *58*, 19021.

[16] X. Y. Yu, H. Gong, B. Gao, X. L. Fan, P. Li, X. L. Huang, K. Chang, Tao Wang, J. P. He, *Chem. Eng. J.* **2022**, *449*, 137774.

[17] G. Y. Qiao, D. H. Guan, S. Yuan, H. Rao, X. Chen, J. A. Wang, J. S. Qin, J. J. Xu, J. H. Yu, *J. Am. Chem. Soc.* **2021**, *143*, 14253.

[18] R. D. Fan, Y. Z. Wu, H. P. Xie, Y. L. Gao, L. Wang, B. Zhao, D. Li, S. C. Liu, Y. Zhang, H. Kong, Y. J. Li, Q. Chen, A. Y. Cao, H. P. Zhou, *ChemSusChem* **2022**, *9*, e202201473.

[19] F. Li, M. L. Li, H. F. Wang, X. X. Wang, L. J. Zheng, D. H. Guan, L. M. Chang, J. J. Xu, Yu Wang, *Adv. Mater.* **2022**, *34*, e2107826.

[20] X. X. Wang, D. H. Guan, F. Li, M. L. Li, L. J. Zheng, J. J. Xu, *Adv. Mater.* **2022**, *34*, e2104792.
